# Fibroblast Activation Protein-Targeted CAR-T Cells Induce Apoptosis in Murine Cardiac Myofibroblasts

**DOI:** 10.1155/cdr/7230505

**Published:** 2025-09-13

**Authors:** Hao Li, Qi Zheng, Yongliang Jiang, Lin Yang, Shuangxiu Li, Ping Yang, Gaosheng Yin, Lin Sun

**Affiliations:** ^1^Department of Cardiology, The Second Affiliated Hospital, Kunming Medical University, Kunming, China; ^2^Yunnan Key Laboratory of Stem Cell and Regenerative Medicine, School of Rehabilitation, Kunming Medical University, Kunming, China

**Keywords:** chimeric antigen receptor T cells, fibroblast activation protein, myocardial fibrosis

## Abstract

Myocardial fibrosis is a common pathological feature in many cardiovascular diseases, yet effective targeted therapies remain elusive. Given the emerging potential of chimeric antigen receptor T (CAR-T) cell therapy in nononcological diseases and fibroblast activation protein (FAP) as a promising target, we engineered a second-generation FAP-targeted CAR construct incorporating the 4-1BB costimulatory domain to enhance therapeutic safety. Using two delivery approaches—lentiviral vectors and lipid nanoparticles (LNPs)—we generated FAP-CAR–engineered Jurkat cells as a preliminary screening model and evaluated their CAR expression, target recognition, and in vitro cytotoxic activity. These engineered cells selectively recognized and induced apoptosis in FAP-expressing cardiac myofibroblasts without triggering excessive IL-6 secretion, supporting their potential for fibrosis-selective cytotoxicity. Our findings provide key preliminary in vitro evidence supporting the design and target-specific functionality of FAP-targeted CAR constructs incorporating the 4-1BB domain, warranting further investigation in primary T cell models for cardiac fibrosis therapy.

## 1. Introduction

Myocardial fibrosis, a pathological hallmark of various cardiac injuries, contributes significantly to the progression of heart failure [1] and remains therapeutically challenging [2]. In response to pathological stimuli, cardiac fibroblasts transdifferentiate into myofibroblasts (myoFbs) through multiple mechanisms such as neuroendocrine activation (e.g., angiotensin II [Ang II]) [3] and profibrotic cytokine signaling (e.g., transforming growth factor-beta [TGF-*β*]) [4]. These activated myoFbs drive excessive extracellular matrix (ECM) deposition that initially supports tissue repair but ultimately disrupts cardiac function through pathological remodeling [5]. Given their central role in myocardial fibrosis [6], targeting myoFbs has emerged as a promising therapeutic strategy [7, 8].

Chimeric antigen receptor (CAR) T cell therapy has demonstrated notable clinical success in oncology [9] and shows emerging promise for nononcological diseases [10–14]. CAR molecules (CARs) are engineered chimeric constructs in which the extracellular recognition domain of the T-cell receptor is replaced with an antibody-derived single-chain variable fragment (scFv) [15]. Upon recognizing specific antigens, CAR-T cells initiate cytotoxic activity through cytokine-mediated mechanisms, inducing target cell elimination [16].

However, identifying specific markers on myoFbs is challenging due to their diverse origins [17]. Interestingly, a study using RNA-seq of heart failure patients' left ventricles revealed upregulated fibroblast activation protein (FAP) [10]. Its selective expression in myoFbs suggests potential therapeutic applications for fibrosis [18]. Additionally, FAP-targeted CAR-T cells, which have been applied in solid tumors [19], may mitigate fibrosis by eliminating myoFbs.

Second-generation CARs, which include costimulatory domains such as CD28 or 4-1BB, have shown improved performance. CD28-based second-generation CARs, studied in myocardial fibrosis [10, 11], offer high cytotoxicity and cytokine secretion but a limited lifespan. In contrast, 4-1BB-engineered CAR-T cells exhibit enhanced persistence with reduced cytokine release [20], making them a safer option for chronic conditions such as fibrosis. Accordingly, we designed a second-generation CAR incorporating the 4-1BB costimulatory domain to improve both safety and therapeutic efficacy in myocardial fibrosis.

CAR-T cell engineering relies on gene delivery methods. Lentiviral vectors are commonly used [21] but are limited by packaging constraints and complex manufacturing. Nonviral alternatives like mRNA-based platforms [22] enable streamlined, cost-effective production. mRNA-based delivery—especially via lipid nanoparticles (LNPs) [23]—offers a nonintegrating, transient alternative with improved safety and production flexibility.

**Scheme 1:**
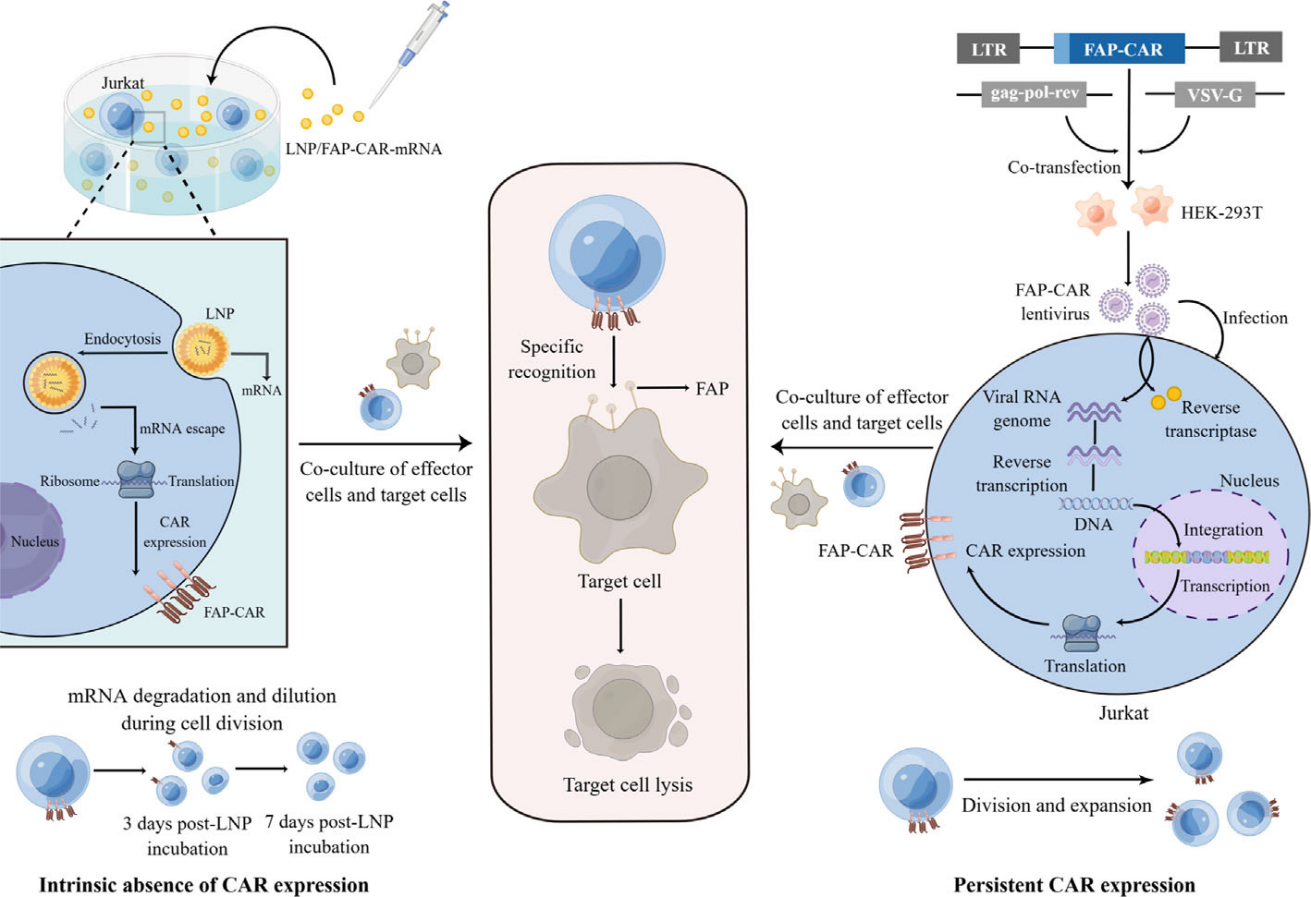
Diagram of in vitro transduction of CAR-Jurkat cells with lentiviral and LNP techniques and subsequent identification and depletion of particular cells. (This diagram is drawn by Figdraw.)

Given the emerging potential of CAR-T therapy in nononcological diseases and FAP as a therapeutic target for myocardial fibrosis, we engineered a second-generation FAP-targeted CAR with the 4-1BB costimulatory domain to enhance safety and persistence. Using dual vector strategies—lentivirus and LNPs—we generated FAP-CAR-Jurkat cells as an initial in vitro screening platform. This model enabled efficient evaluation of CAR expression, antigen-specific cytotoxicity, and cytokine secretion in a standardized setting. While Jurkat cells cannot fully recapitulate the function of primary T cells, they provided a rapid and reproducible system for preliminary validation. These findings establish foundational proof-of-concept for future studies using primary T cells in myocardial fibrosis intervention (Scheme 1).

## 2. Materials and Methods

### 2.1. Cell Lines

The mouse cardiac fibroblasts (MCFs; BNCC 340098) and human T lymphocyte cells (Jurkat; Clone E6-1, ATCC TIB-152) cell lines were grown in RPMI-1640 (GIBCO) containing 10% fetal bovine serum (FBS; GIBCO) and 1% penicillin–streptomycin (Biosharp). HEK-293T cells (ATCC CRL-3216) were cultured in high-glucose DMEM (Servicebio) supplemented with 10% FBS and 1% penicillin–streptomycin (Biosharp). All cells were cultured at 37°C with 5% CO_2_.

FAP-expressing HEK-293T cells (FAP-293T) were generated by transducing cells with a lentiviral vector (GeneCopoeia) coexpressing murine FAP and eGFP, linked by an internal ribosome entry site (IRES) element, followed by puromycin (3 *μ*g/mL) selection to establish stable expression. FAP overexpression was confirmed by fluorescence microscopy, flow cytometry, and Western blotting. Nontransduced HEK-293T cells (NC) were used as the control group.

### 2.2. Reagents and Antibodies

Ang II (A800620) and TGF-*β* (HY-P70648) were purchased from MedChemExpress (Shanghai, China). Cholesterol (C6213) and DSPC (D875630) were obtained from Macklin (Shanghai, China), while DOTAP (132172-61-3) and DSPE-PEG2000-NH2 (474922-26-4) were sourced from AVT (Shanghai, China). Lenti-X Concentrator and RetroNectin were purchased from TaKaRa (Tokyo, Japan). Alexa Fluor 647F(ab⁣′)2 IgG (115-606-003) was from Jackson ImmunoResearch (West Grove, PA). CD247 rabbit polyclonal antibody (12837-2-AP), Collagen Type I monoclonal antibody (66761-1), and Collagen Type III polyclonal antibody (22734-1) were obtained from Proteintech (Wuhan, China). Mouse FAP*α* antibody was sourced from R&D Systems (Minneapolis, MN, United States). Antibodies including rabbit FAP1 (AF5344), mouse vimentin (BF8006), mouse alpha-SMA (BF9212), rabbit GAPDH (AF7021), rabbit Bax (AF0120), rabbit Bcl-2 (AF6139), rabbit Caspase 3 (AF6311), goat anti-rabbit IgG HRP (S0001), and goat anti-mouse IgG HRP (S0002) were from Affinity Biosciences (Jiangsu, China). Rabbit *β*-actin (AC038) was acquired from ABclonal Biotechnology. Rabbit Alexa Fluor 647 goat IgG isotype (403006) was acquired from BioLegend (San Diego, CA).

### 2.3. Generation of Mouse myoFbs

Proliferating MCFs were passaged into culture dishes. When cultures reached 50%–60% confluency, cells were treated with Ang II or TGF-*β* to induce fibrotic activation. Ang II treatment: The medium was replaced with complete medium (RPMI‐1640 + 10% FBS) containing Ang II after PBS rinse. TGF-*β* treatment: The medium was replaced with complete medium (RPMI‐1640 + 10% FBS) containing TGF-*β* after PBS rinse. Control group: The medium was replaced with complete medium (RPMI‐1640 + 10% FBS) without additives. Cells were incubated for 48 h to generate myoFbs.

### 2.4. FAP-CAR Vector Transduction and Analysis

Two vectors—lentiviral and LNPs—were developed for CAR-Jurkat cells generation. Full-length CAR DNA was synthesized by GenScript and cloned into the pCDH-EF1-MCS vector to create the FAP-CAR-pCDH-EF1-MCS plasmid. This plasmid, along with psPAX2 and pMD2.G packaging plasmids, was cotransfected into HEK-293T cells for lentiviral production. Viral supernatants were collected at 48 and 72 h, concentrated with Lenti-X Concentrator. For LNP vector synthesis, CAR mRNA was synthesized, purified, and encapsulated in LNPs composed of DOTAP, DSPC, cholesterol, and DSPE-PEG2000-NH2 and characterized by transmission electron microscopy (TEM) and dynamic light scattering (DLS).

For lentiviral transduction, plates were first coated with RetroNectin solution and incubated overnight at 4°C. After removing the solution, wells were blocked with sterile 2% BSA for 30 min at room temperature (RT), followed by a PBS wash. Lentiviral particle suspension was added, and plates were centrifuged at 1200 × g for 2 h at 32°C to enhance viral binding. The supernatant was discarded, and target cells were added for incubation. LNPs, confirmed to be nontoxic, were used to transduce Jurkat cells by incubation with a nontoxic LNP concentration for 48 h, generating FAP-CAR-Jurkat cells.

After 48 h of transduction with lentivirus or LNPs, 2 × 10^5^ cells from both the control and transduced groups were collected, washed twice with PBS, and centrifuged. The pellet was resuspended in PBS, and Alexa Fluor 647 AffiniPure F(ab⁣′)_2_ IgG antibody was added per the manufacturer's instructions. Cells were incubated on ice for 20–30 min in the dark, then washed twice with PBS, and resuspended in 500 *μ*L of PBS. The suspension was filtered through a 200-mesh nylon filter and analyzed by flow cytometry, with blank and isotype controls for gating.

### 2.5. In Vitro Cytotoxicity Assay (Lactate Dehydrogenase [LDH] Release)

FAP-293T cells were generated by lentiviral transduction of FAP as described (Section 2.1). Target cells included FAP-293T cells, Ang II or TGF-*β* induced myoFbs (Section 2.3), NC, and untreated MCFs (nonactivated controls). CAR-Jurkat or nontransduced control Jurkat (NC-Jurkat) cells (non-CAR-transduced) were cocultured with target cells at effector-to-target (E:T) ratios of 1:1, 5:1, and 10:1 in triplicate 12 or 96-well plates. After 24 h, supernatants were collected for IFN-*γ* and IL-6 quantification via ELISA. Cytotoxicity was assessed by LDH release.

Cytokine levels in supernatants were quantified using commercial sandwich ELISA kits (IFN-*γ*, MEIMIAN, MM-0033H2; IL-6, MEIMIAN, MM-0049H2) according to the manufacturer's instructions. Briefly, 96-well plates precoated with capture antibodies were incubated with supernatants or standards (diluted in assay buffer) for 2 h at RT. After washing, biotinylated detection antibodies were added for 1 h, followed by streptavidin-HRP (30 min, RT). TMB substrate was used for colorimetric detection (OD450 nm). Standard curves were generated using recombinant cytokines, and sample concentrations were interpolated from linear regression curves (triplicate measurements).

CAR-Jurkat cell cytotoxicity was assessed using a LDH release assay (Beyotime, C0017). CAR-Jurkat or NC-Jurkat and target cells were cocultured at specified E:T ratios for 24 h. Supernatants were mixed with LDH substrate (30 min, dark), and absorbance (490 nm) was measured. Controls included a spontaneous condition (targets alone) and a maximum condition (targets +1% Triton X-100). Cytotoxicity (%) was calculated as (experimental − spontaneous)/(maximum − spontaneous) × 100.

### 2.6. Western Blot Analysis

Total protein was extracted and quantified using the BCA Protein Assay Kit (Beyotime). Proteins were separated by SDS-PAGE, transferred to PVDF membranes, and blocked with 5% nonfat milk in TBST for 2 h at RT. The membranes were then incubated with primary antibodies diluted in TBST under gentle agitation at 4°C overnight, followed by incubation with horseradish peroxidase–conjugated secondary antibodies—anti-rabbit (1:10,000, S0001, Affinity Biosciences) or anti-mouse (1:10,000, S0002, Affinity Biosciences)—for 1 h at RT. The following primary antibodies were used: CD247 (rabbit, 1:1500, 12837-2-AP, Proteintech), FAP1 (rabbit, 1:1000, AF5344, Affinity Biosciences), vimentin (mouse, 1:2000, BF8006, Affinity Biosciences), *α*-smooth muscle actin (*α*-SMA) (mouse, 1:2000, BF9212, Affinity Biosciences), Collagen Type I (mouse, 1:3000, 66761-1-Ig, Proteintech), Collagen Type III (rabbit, 1:1000, 22734-1-AP, Proteintech), Bax (rabbit, 1:2000, AF0120, Affinity Biosciences), Caspase 3 (rabbit, 1:1500, AF6311, Affinity Biosciences), Bcl-2 (rabbit, 1:1500, AF6139, Affinity Biosciences), GAPDH (mouse, 1:5000, AF7021, Affinity Biosciences), and *β*-actin (rabbit, 1:30000, AC038, ABclonal Biotechnology). Protein bands were visualized using an ECL reagent (Proteintech) and detected with a chemiluminescence imaging system (GE Healthcare). Band intensities were quantified using ImageJ software, and relative protein expression levels were normalized to GAPDH or *β*-actin.

### 2.7. Immunofluorescence Staining

Cells (control, Ang II, or TGF-*β*-treated) were fixed with 4% paraformaldehyde (15 min), permeabilized with 0.1% Triton X-100 (10 min), and blocked with 5% BSA (1 h). Primary antibodies *α*-SMA (mouse, 1:200, BF9212, Affinity Biosciences), FAP (rabbit, 1:200, A23789, ABclonal), and vimentin (mouse, 1:200, BF8006, Affinity Biosciences) were incubated overnight (4°C), followed by Alexa Fluor–conjugated secondary antibodies (1:500, 1 h, RT). Nuclei were counterstained with DAPI. Images were captured using a fluorescence microscope.

### 2.8. Cell Counting Kit-8 (CCK8) Assay for T Cell Proliferation

To assess the proliferative capacity of engineered CAR-T cells, CAR-Jurkat cells and negative control-Jurkat cells were seeded in 96-well flat-bottom plates at a density of 1 × 10^4^ cells per well in 100 *μ*L of complete RPMI-1640 medium. On Days 1–5, 10 *μ*L of CCK8 solution (Beyotime, C0038) was added to each well and incubated for 2 h at 37°C. Absorbance was measured at 450 nm using a microplate reader.

### 2.9. CCK8 Assay for LNP Cytotoxicity

To evaluate the cytotoxicity of LNPs, Jurkat T cells were seeded in 96-well plates at 1 × 10^4^ cells per well and treated with increasing concentrations of LNPs (0, 1.25, 2.5, 5, 10, and 20 *μ*g/mL). After 24 h of incubation at 37°C, 10 *μ*L of CCK8 reagent was added to each well, followed by a 2-h incubation. Absorbance at 450 nm was measured to determine cell viability.

### 2.10. TEM

Synthesized LNPs were diluted in ultrapure water and deposited onto carbon-coated copper grids (10 *μ*L, 1 min adsorption). Excess liquid was blotted, and grids were negatively stained with 2% phosphotungstic acid (PTA, pH 7.0) for 30 s. After air-drying, samples were imaged using a JEOL JEM-1400Plus TEM operated at 120 kV. Size distribution was analyzed from ≥ 100 particles (ImageJ v1.53).

### 2.11. DLS

The hydrodynamic diameter, polydispersity index (PDI, a dimensionless parameter reflecting sample homogeneity; PDI < 0.2 indicates monodisperse particles), and zeta potential of LNPs were measured using a Zetasizer Nano ZS90 (Malvern Panalytical, United Kingdom). Samples were dispersed in deionized water (0.1 mg/mL), sonicated for 3 min, and loaded into disposable polystyrene cuvettes. Measurements were performed at 25°C with a 633 nm laser, 90° scattering angle, and automatic attenuator optimization. Data were acquired as 10 runs (6 s each) per sample, averaged over three independent replicates. Correlation curves were inspected to exclude aggregation.

### 2.12. Statistical Analyses

Data were confirmed to follow a normal distribution. Differences between two groups were assessed using Student's *t*-test, while comparisons among multiple groups were performed using one-way ANOVA followed by Tukey's post hoc test. All experiments were independently repeated at least three times (biological replicates), and results are presented as mean ± SD. Statistical significance was defined as *p* < 0.05.

## 3. Result

### 3.1. Engineering a Second-Generation FAP-Targeting CAR: Lentiviral Expression Vector Construction and mRNA Synthesis

**Figure 1:**
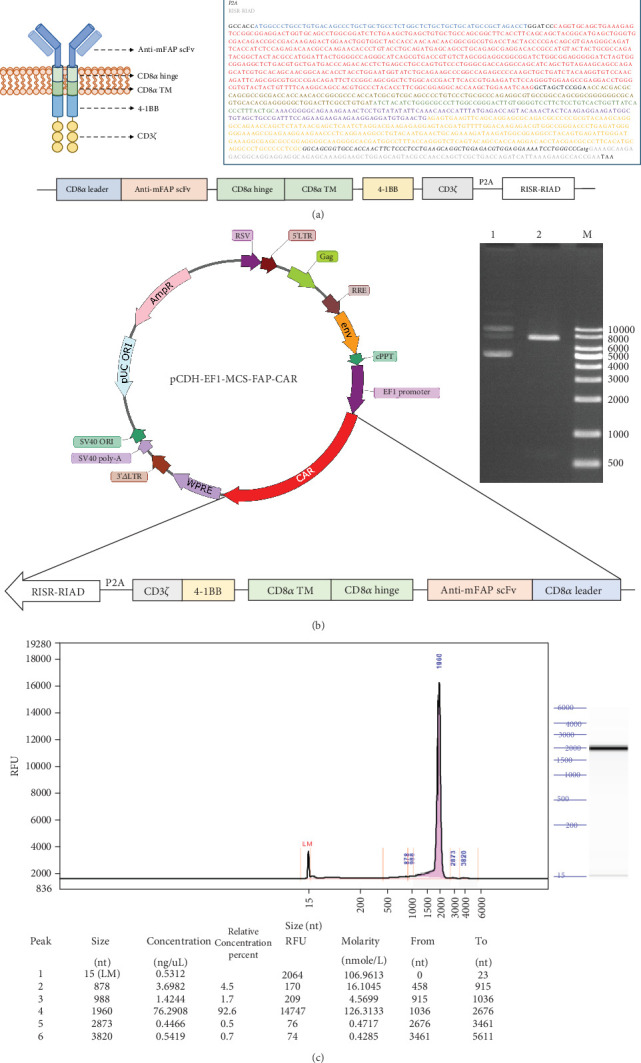
(a) Schematic representation of the FAP-CAR molecular structure on the cell membrane (left). Nucleic acid sequence of the second-generation FAP-CAR, with each component highlighted in a distinct color (right). (b) Schematic of the recombinant vector pCDH-EF1-MCS-FAP-CAR (left). Electrophoresis of the plasmid after double digestion with EcoRI and KpnI (right): Lane 1, undigested plasmid; Lane 2, digested plasmid; Lane M, DNA ladder (bp). (c) FAP-CAR mRNA quality control, showing 92.6% purity.

To evaluate the ability of CAR-T cells to target and eliminate FAP-expressing myoFbs, we engineered a second-generation FAP-targeting CAR. This construct comprises an scFv derived from a murine anti-FAP monoclonal antibody (clone 73.3), linked to a human CD8*α* chain forming the hinge and transmembrane regions, and an intracellular domain containing the human 4-1BB and CD3*ζ* signaling components (Figure 1a). Additionally, a Regulatory Subunit I Anchor Disturber (RIAD) peptide was incorporated to enhance resistance to adenosine and prostaglandin E2-mediated immunosuppression, thereby improving CAR-T cell efficacy in suppressive environments [24].

We cloned the FAP-CAR nucleic acid sequence into the lentiviral vector pCDH-EF1-MCS and validated the construct via double enzyme digestion (EcoRI and KpnI) (Figure 1b). The resulting pCDH-EF1-MCS-FAP-CAR plasmid was confirmed (OD260/280 = 1.93). Additionally, we synthesized and purified the FAP-CAR mRNA, which featured 101 poly A tails and a cap structure, achieving a high purity of 92.6% (OD260/280 > 1.8 and OD260/OD230 > 2.0) (Figure 1c).

### 3.2. Packaging of FAP-CAR Lentiviral Particles and Synthesizing of LNPs Loaded With FAP-CAR mRNA

**Figure 2:**
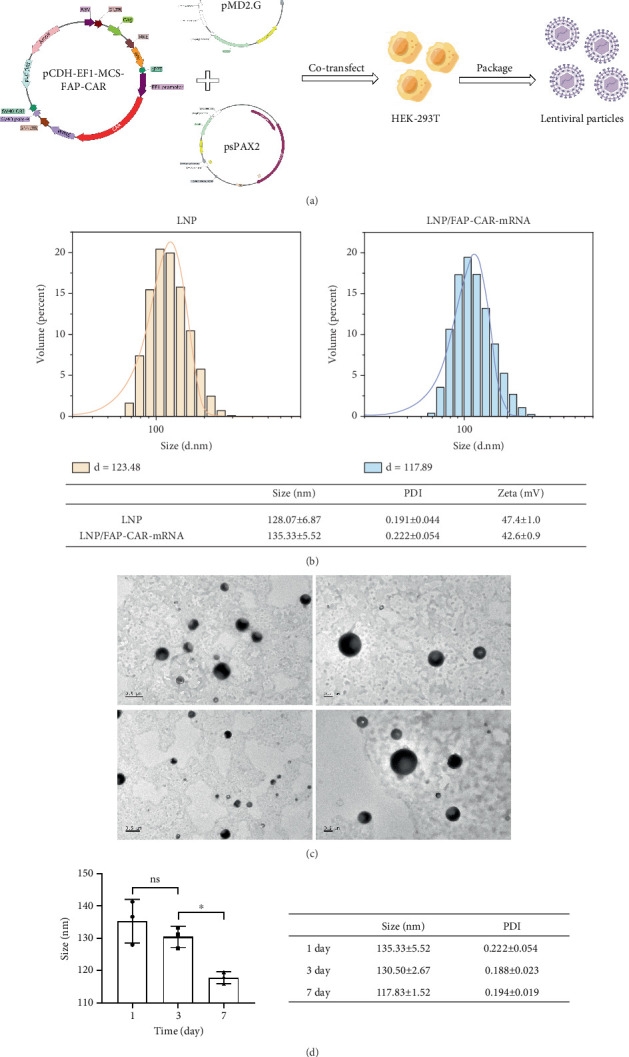
(a) Schematic of lentiviral packaging. (b) Dynamic light scattering (DLS) analysis of LNP size, PDI, and zeta potential. (c) Transmission electron microscopy (TEM) analysis of LNP morphology and size. (d) Stability assessment of LNPs (*n* = 3 for each group) (⁣^∗^*p* < 0.05, and ns indicates no significant difference).

The recombinant expression plasmid pCDH-EF1-MCS-FAP-CAR was cotransfected into HEK-293T cells along with lentiviral packaging plasmids psPAX2 and pMD2.G to obtain lentiviral particles (Figure 2a). The viral suspension was concentrated and then evaluated for lentivirus concentration by measuring HIV-1 P24 antigen content using an ELISA assay (Figure S1 and Table 1).

We synthesized empty LNPs and LNPs loaded with FAP-CAR mRNA using the thin-film hydration method (Table 2). DLS analysis (Figure 2b) showed that both the empty and mRNA-loaded LNPs had particle sizes under 200 nm, with the latter being slightly larger due to mRNA loading. Both LNPs exhibited a PDI (a dimensionless parameter reflecting particle size distribution; PDI < 0.2 indicates monodispersity) around 0.2, indicating good homogeneity, and Zeta potentials above +40 mV. TEM images confirmed that the LNPs were spherical (Figure 2c). LNPs were stored at 4°C, and their particle sizes and PDIs were measured on Days 1, 3, and 7 to assess stability. The results indicated minimal variation in particle size over the first 3 days, suggesting that the LNPs remain stable at 4°C for short-term storage and should be used soon after synthesis (Figure 2d).

### 3.3. FAP-CAR Lentivirus Effectively Transduced Both HEK-293T and Jurkat Cells

To validate the functionality of the synthesized FAP-CAR lentivirus, we first transduced HEK-293T cells—a model system with high lentiviral susceptibility—to confirm CAR expression and viral efficacy before applying the lentivirus to Jurkat cells. Western blot analysis of transduced HEK-293T cells revealed robust CAR protein expression compared to nontransduced controls (Figure S2A). SnapGene software was used to translate the CAR nucleic acid sequence into protein fragments, showing a full-length protein of 621 amino acids (~68 kDa) (Figure S2B). Flow cytometry further demonstrated CAR surface expression in > 40% of transduced 293T cells (Figure S2C), confirming lentiviral functionality.

**Figure 3:**
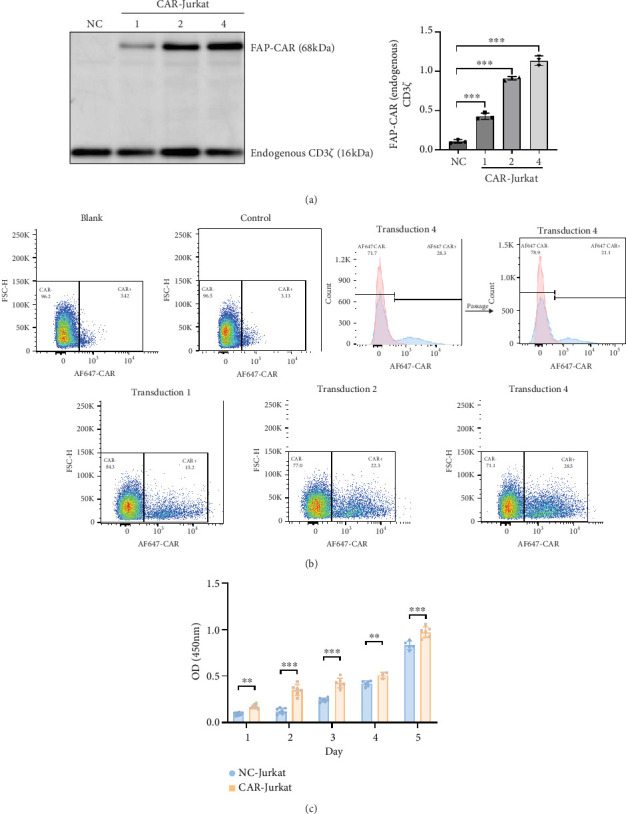
(a) Western blot analysis of CAR protein expression in Jurkat cells postlentiviral transduction across different groups using endogenous CD3*ζ* as an internal control (*n* = 3 for each group). Given that Jurkat cells naturally express stable levels of endogenous CD3*ζ* on their surface, this can serve as an internal control. (b) Flow cytometry assessment of CAR surface expression in transduced Jurkat cells from various groups (blank, blank control; control, CAR staining control; isotype, isotype control; transduction, transduction group). Flow cytometry comparison of CAR expression in CAR-Jurkat cells from Transduction Group 4 before and after 10 passages of expansion culture. (c) CCK8 assay evaluating the proliferative activity of nontransduced and transduced Jurkat cells over 5 days; higher OD values indicate greater proliferation (*n* ≥ 3 for each group) (⁣^∗∗^*p* < 0.01 and ⁣^∗∗∗^*p* < 0.001).

We then transduced Jurkat cells with the validated FAP-CAR lentivirus at escalating concentrations (9711.37, 19422.74, and 38845.48 pg/mL). Western blot analysis showed significant CAR protein expression in all transduced groups compared to controls, with expression levels positively correlating with lentiviral concentration (Figure 3). Flow cytometry confirmed surface CAR expression across all transduced groups, with the highest positive rate (28.5%) in the highest concentration group (Figure 3). These results demonstrate successful transduction of Jurkat cells. The group with the highest transduction efficiency was selected for expansion. CCK8 assays indicated enhanced proliferation in CAR-Jurkat cells compared to controls, likely due to the inclusion of costimulatory domains (Figure 3). After over 10 passages, the CAR-positive rate remained stable in the highest transduction group (Figure 3b, right), suggesting stable long-term CAR expression in these cells.

### 3.4. LNPs Loaded With FAP-CAR mRNA Successfully Transduced Jurkat Cells

**Figure 4:**
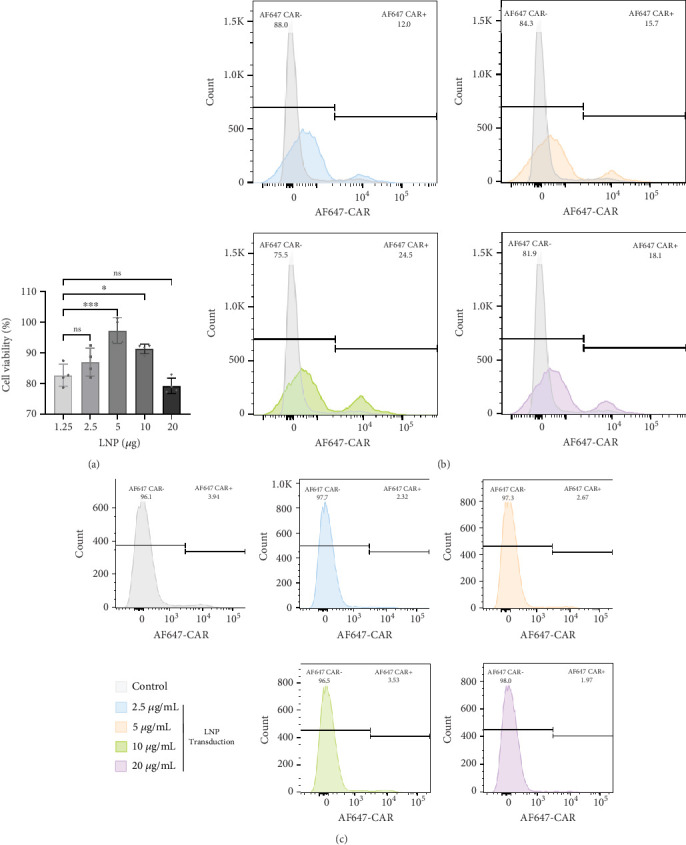
(a) CCK8 cytotoxicity assay evaluated the toxicity of LNPs/FAP-CAR-mRNA on Jurkat cells (*n* = 4 for each group). (b) Flow cytometry assessed CAR expression rates in Jurkat cells transduced with LNPs/FAP-CAR-mRNA. (c) Flow cytometry reexamined CAR expression in transduced Jurkat cells after more than 7 days of expansion (⁣^∗∗^*p* < 0.01, and ns indicates no significant difference).

A key component of the LNPs synthesized in this study is the cationic lipid DOTAP, widely used in delivery systems [25]. Notably, DOTAP forms the basis of EndoTAG-1, a paclitaxel-encapsulated cationic liposome currently in Phase III clinical trials [26]. However, DOTAP's positive charge can be toxic to normal tissues if too high, making it essential to assess the LNPs' safety. The cytotoxicity of the synthesized LNPs was evaluated by measuring Jurkat cell survival after incubation with LNPs using the CCK8 assay. Results showed that LNPs (1.25–10 *μ*g/mL) maintained Jurkat cell viability > 80%, while 20 *μ*g/mL reduced viability to < 80% (Figure 4a), suggesting mild cytotoxicity at the highest dose. Jurkat cells (5 × 10^5^) were incubated with 2.5, 5, 10, and 20 *μ*g/mL of LNPs/FAP-CAR-mRNA for 48 h. Flow cytometry of CAR-Jurkat cells showed dose-dependent CAR expression from 2.5 to 10 *μ*g/mL LNPs. However, at 20 *μ*g/mL, CAR expression decreased (Figure 4b), potentially due to higher LNPs cytotoxicity at this concentration. LNPs allowed FAP-CAR mRNA to escape into the cytoplasm for translation, resulting in transient expression without genomic integration. After more than 7 days of expansion, flow cytometry revealed that CAR expression was nearly undetectable in all groups, indicating loss of CAR molecules during cell division and expansion (Figure 4c).

### 3.5. Generated Two Types of Target Cells: FAP-Expressing 293T Cells and myoFbs

**Figure 5:**
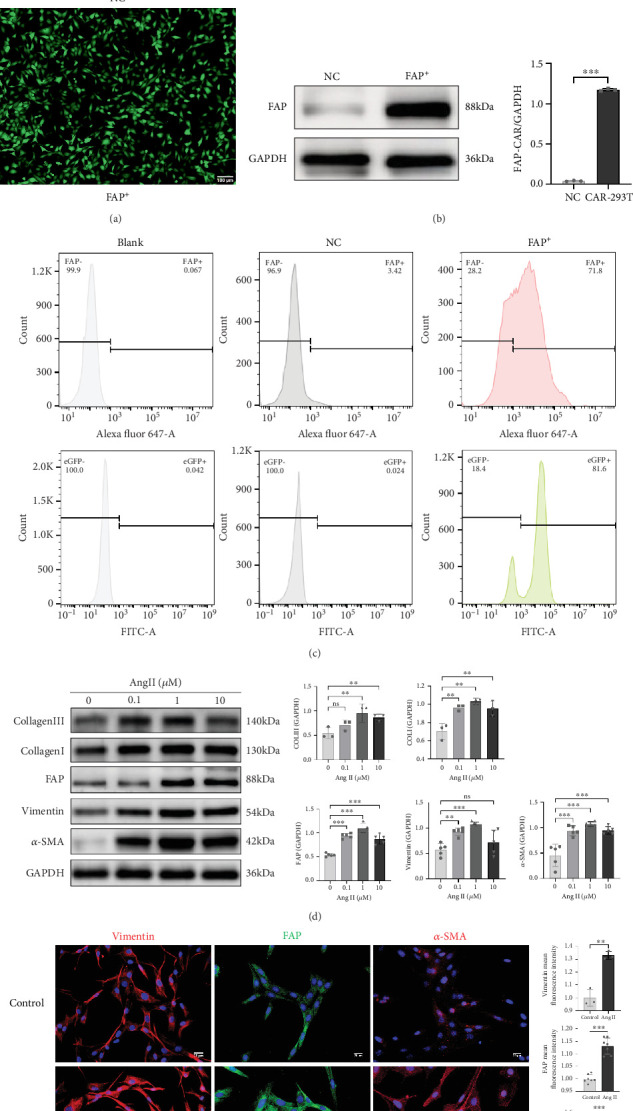
(a) Fluorescence microscopy (10×) of FAP-293T cells postpuromycin screening showed eGFP (green) expression in successfully transduced cells. (b) Western blot analysis of FAP protein expression in FAP-293T cells postscreening (*n* = 6 for each group). (c) Flow cytometry analysis of eGFP and FAP expression in FAP-293T cells after multiple screening generations (blank, blank control group; NC, untransduced staining group; FAP^+^, transduced staining group). (d) Western blot analysis of fibrosis marker proteins in MCFs under different Ang II concentrations (*n* ≥ 3 for each group). (e) Immunofluorescence detection of vimentin, FAP, and *α*-SMA in MCFs after 48 h of 1 *μ*M Ang II treatment (*n* ≥ 3 for each group) (⁣^∗^*p* < 0.05, ⁣^∗∗^*p* < 0.01, and ⁣^∗∗∗^*p* < 0.001, and ns represents no statistical difference).

HEK-293T cells were stably transduced with a lentiviral vector encoding murine FAP and eGFP, followed by selection with 3 *μ*g/mL puromycin. Fluorescence microscopy revealed > 90% eGFP-positive cells (Figure 5a), and Western blot confirmed markedly elevated murine FAP protein expression compared to controls (Figure 5b). Flow cytometry analysis showed 81.6% of cells expressing eGFP, with 71.8% coexpressing FAP on the surface (Figure 5c), confirming the successful generation of a murine FAP-overexpressing HEK-293T cell line (termed FAP-293T) for downstream CAR-T targeting studies.

To induce fibrotic activation of cardiac fibroblasts, MCFs were treated with either Ang II (0, 0.1, 1, and 10 *μ*M) or TGF-*β* (0, 5, 10, and 20 ng/mL) for 48 h. Western blot analysis showed a dose-dependent upregulation of fibrosis-associated proteins, including *α*-SMA, vimentin, FAP, Collagen I, and Collagen III in response to Ang II stimulation, with peak expression observed at 1 *μ*M (Figure 5d). Consistently, immunofluorescence staining confirmed increased vimentin, FAP, and *α*-SMA expression in the 1 *μ*M Ang II group (Figure 5e). Similarly, treatment with TGF-*β* led to increased fibrosis-associated protein expression, with the most robust changes seen at 10 ng/mL (Figure S4A). Immunofluorescence staining confirmed increased *α*-SMA and FAP expression in the 10 ng/mL TGF-*β* group (Figure S4B). These findings demonstrate that both Ang II and TGF-*β* treatments can effectively induce fibroblast-to-myoFb transition. Based on the protein expression levels, 1 *μ*M Ang II and 10 ng/mL TGF-*β* were selected for subsequent experiments to induce fibrotic activation of MCFs and upregulate FAP expression, allowing evaluation of CAR-Jurkat cell targeting efficiency.

### 3.6. CAR-Jurkat Cells Exhibit Good Targeting and Killing Ability Against FAP-293T Cells and myoFbs

**Figure 6:**
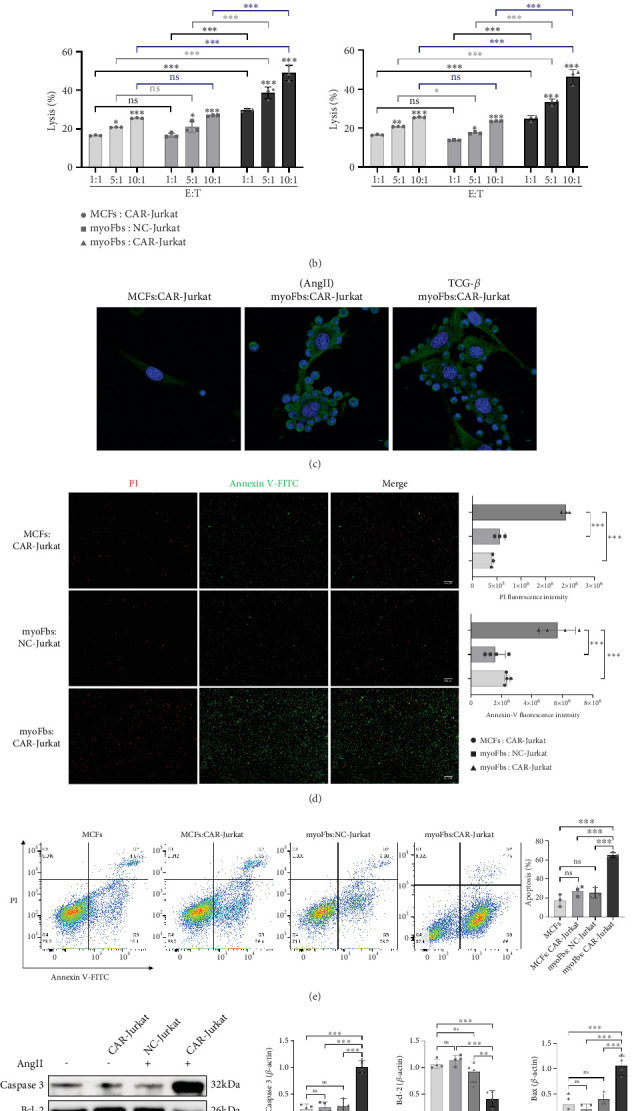
(a) ELISA assays measured cytokine secretion (IFN-*γ* and IL-6) after coculture (*n* = 3 for each group). (b) Target cell lysis quantified by LDH release assay at indicated E:T ratios (*n* = 3 for each group). (c) Confocal microscopy showing CAR-Jurkat cells (blue, DAPI) specifically clustering around FAP^+^ myoFbs (green, FAP; blue, DAPI). Apoptosis quantification by (d) fluorescent microscopy (Annexin V-FITC/PI) and (e) flow cytometry (Annexin V-FITC [green] and PI [red] staining). Early apoptotic cells were FITC-positive, while late apoptotic cells were double-stained. (f) Western blot analysis of apoptosis-related proteins in target cells after 24 h coculture (⁣^∗^*p* < 0.05, ⁣^∗∗^*p* < 0.01, and ⁣^∗∗∗^*p* < 0.001, and ns represents no statistical difference).

CAR-Jurkat cells, stably expressing a FAP-specific CAR via lentiviral transduction, or NC-Jurkat cells, were cocultured with target cells at varying E:T ratios for 24 h. Target cells included FAP-293T cells, Ang II- or TGF-*β*-induced myoFbs, untreated MCFs, and NC. CAR-T cells eliminate target cells primarily via cytokine secretion, such as IFN-*γ*, while excessive IL-6 secretion is associated with cytokine release syndrome (CRS), a systemic inflammatory response associated with immune cell overactivation [27]. As shown in Figure 6a, ELISA results demonstrated a significant, E:T ratio-dependent increase in IFN-*γ* secretion by CAR-Jurkat cells when cocultured with myoFbs. At a 10:1 ratio, IFN-*γ* levels were significantly higher than those in cocultures of CAR-Jurkat with MCFs or NC-Jurkat with myoFbs, indicating specific activation. In contrast, IL-6 levels remained below 25 ng/L in all groups, suggesting no risk of CRS. Consistently, LDH release assays revealed enhanced cytotoxicity by CAR-Jurkat cells in a ratio-dependent manner, peaking at 10:1 and significantly exceeding levels in control groups (Figure 6b). Confocal imaging (Figure 6c) showed clustering of CAR-Jurkat cells around FAP-positive myoFbs, but not around FAP-negative MCFs, indicating specific recognition. Similar results were observed in cocultures with FAP-293T versus 293T cells (Figure S5). These findings confirm that CAR-Jurkat cells mediate target-specific lysis without detectable nonspecific killing, demonstrating precise antigen-dependent cytotoxicity while maintaining a favorable safety profile. Apoptosis staining by fluorescence microscopy and flow cytometry confirmed significant apoptosis in FAP-positive myoFbs, but not in MCFs (Figure 6d,e). Western blot analysis further revealed that CAR-Jurkat coculture reduced Bcl-2 levels and increased Bax and Caspase 3 expression (Figure 6f), suggesting apoptosis as a mechanism of cytotoxicity.

## 4. Discussion

CAR-T cell therapy, a leading cellular treatment, has shown promising results in hematological cancers. Studies from the University of Pennsylvania in 2019 [10] and 2022 [11] demonstrated that CAR-T cells can effectively target and eliminate FAP-expressing myoFbs, thereby inhibiting myocardial fibrosis. These studies suggest that CAR-T cell therapy has been explored in cardiovascular diseases, offering new research avenues for conditions like myocardial fibrosis, which lack effective treatments. Continuing with FAP as the target, we investigated the potential of CAR-T cells in treating myocardial fibrosis, expecting further evidence to support CAR-T cell therapy as a viable strategy for its inhibition.

Compared to the success in hematological tumors, the application of CAR-T cell therapy in cardiovascular diseases has greater challenges. The durability and expansion of CAR-T cells are strongly correlated with the therapeutic effectiveness. The structure of CAR molecules has significant importance in these aspects. Second-generation CAR molecules contain an additional costimulatory domain and exhibit a higher proliferation rate and cytotoxicity than the first generation, thus being widely used. In the studies on myocardial fibrosis–related CAR-T cell therapy, the second-generation CAR with the CD28 costimulatory domain was used exclusively [10, 11]. The CD28 CAR-T cells have potent toxicity, high cytokine production, but short half-lives. In contrast, CAR-T cells with the 4-1BB costimulatory domain exhibit lower cytokine secretion but greater in vivo expansion and persistence [20]. The CAR construct evaluated in this preliminary Jurkat-based screening incorporates the 4-1BB costimulatory domain. While Jurkat cells have constitutive signaling that limits direct extrapolation to primary T cells, our ELISA results showed low cytokine secretion, including reduced IL-6 levels, and CCK8 assays indicated robust proliferation. These findings align with the reported profile of 4-1BB-based CARs—reduced cytokine release and improved persistence—and provide initial support for its potential safety advantages. Nonetheless, definitive evaluation of 4-1BB's functional impact requires validation in primary T cells and disease-relevant models.

CAR-T cells activate signaling pathways and exert effector functions upon binding to specific antigens on target cells. In hematological cancers, CAR-T cells can efficiently recognize and kill circulating target cells, leading to strong therapeutic outcomes. However, for solid tumors and nontumor diseases, CAR-T cells must infiltrate target tissues through blood circulation to be effective [28]. Limited infiltration of CAR-T cells into target tissues can significantly reduce therapeutic efficacy [29, 30]. To improve the therapeutic effect of CAR-T cells in treating myocardial fibrosis, increasing their infiltration into fibrotic tissue is crucial. Chemokines and their receptors are key mediators of CAR-T cells migration to target tissues. A potential strategy is to incorporate chemokine receptor sequences into CAR constructs to enhance their in vivo migration and infiltration. By analyzing the chemokine profile of myoFbs in fibrotic myocardium and introducing matching chemokine receptors into CAR molecules, CAR-T cell infiltration into fibrotic areas can be improved, potentially boosting therapeutic efficacy.

CAR-T cell therapy can cause notable and manageable side effects, particularly off-target effects, where CAR-T cells attack normal tissues expressing the same antigen as the target cells, leading to tissue damage [31]. In this study, CAR-T cells targeting FAP on myoFbs were developed with strong cytotoxicity. However, since FAP is also expressed in bone marrow stromal cells and osteoblasts, systemic elimination of FAP-positive cells may cause anemia, bone loss, and cachexia [32]. Thus, identifying more specific and safer targets for treating myocardial fibrosis with CAR-T cell therapy is crucial.

Currently, no definitive markers exist for resident cardiac fibroblasts and myoFbs. Early markers like thymocyte differentiation antigen 1 (Thy-1, also known as CD90) [33], fibroblast-specific protein 1 (FSP1, also known as S100A4) [34], platelet-derived growth factor receptor-*α* (PDGFR*α*) [35], and *α*-SMA lack specificity, as they also label other cell types like endothelial cells and immune cells [36]. Periostin (Postn), an 811-amino acid polypeptide secreted by cardiac fibroblasts and myoFbs, is expressed exclusively in adult tissues after injury [37], making it a reliable marker for myoFbs [38]. Postn-expressing myoFbs form protective scars after heart injury. In mice, Postn deletion impairs scar formation [39], indicating its potential as a target for inhibiting myocardial fibrosis. However, as Postn is a secreted protein and CAR-T cells require surface antigens for recognition, it is unsuitable as a CAR-T target despite its specificity.

Research suggests that distinct fibroblast subsets exhibit cell-specific responses to different injury models. In ischemic events such as myocardial infarction, epicardium-derived fibroblasts are key drivers of severe fibrosis in the anterior left ventricular wall. By contrast, endocardium-derived fibroblasts (EndoFb) proliferate more prominently in regions with severe fibrosis following pressure overload. Ablating EndoFb reduces their proliferation, mitigates fibrosis severity, and preserves cardiac function [17]. Identifying surface antigens unique to different fibroblast subsets could enable the design of CAR molecules tailored for CAR-T cell therapy against myocardial fibrosis of varying causes, improving therapeutic precision. In this study, Ang II was used to induce pressure-overload fibrosis in MCFs in vitro. Targeting surface antigens on EndoFb could offer more precise and safer treatments for this form of fibrosis. Further investigation is required to identify specific surface markers on myoFbs.

Most CAR-T cell therapies, whether approved or in clinical trials, rely on viral vectors. However, nonviral vectors hold promise due to their simpler manufacturing, lower costs, and quicker availability. In this study, LNPs were used to deliver FAP-CAR-mRNA, enabling transient CAR expression in Jurkat cells in vitro, which diminished after 7 days of proliferation, limiting the therapy's durability. However, LNPs-mediated transduction was less efficient than lentiviral vectors, likely due to the lack of targeting specificity. Conjugating T-lymphocyte-targeting antibodies to the LNPs surface could improve transduction efficiency and enable in vivo application.

This study has several limitations, foremost being the use of Jurkat cells, an immortalized T cell line with constitutive signaling that does not fully replicate the activation, exhaustion, persistence, or cytokine regulation of primary T lymphocytes. While this model enabled rapid and standardized evaluation of CAR expression, transduction efficiency (lentivirus vs. LNP), and antigen-specific cytotoxicity, it is inherently unsuitable for definitive comparisons of costimulatory domains (e.g., 4-1BB vs. CD28) or prediction of clinical safety profiles such as CRS [40–42]. Our findings, therefore, provide essential proof-of-concept and design validation data but must be interpreted within the constraints of this in vitro screening platform. Future work will focus on generating FAP-targeted CAR-T cells from primary murine splenocytes for in vivo modeling and from human T cells for translational studies. Comprehensive evaluation in these physiologically relevant systems—including cytotoxic efficacy, persistence, cytokine secretion (e.g., IL-6), and safety (e.g., off-target effects, anemia, and bone loss)—is essential to substantiate the therapeutic potential suggested by our Jurkat data. Given that Jurkat-derived CAR-T cells are unsuitable for in vivo use due to immunogenicity, murine T cells will be used in animal models of myocardial fibrosis to further assess efficacy and toxicity. Nonetheless, the Jurkat-based platform remains a valuable tool for preliminary CAR design optimization prior to more resource-intensive primary T cell validation.

## 5. Conclusion

In this study, we used an engineered Jurkat cell model for preliminary in vitro validation of FAP-CAR constructs delivered via lentiviral and LNP methods. Lentiviral vectors provided higher transduction efficiency and stable expression, while LNPs enabled transient expression. The 4-1BB-based FAP-CAR exhibited effective cytotoxicity against FAP-positive myoFbs with minimal IL-6 secretion and no off-target effects. These findings offer initial proof of concept for the CAR design and delivery strategies. However, further validation in primary T cells and relevant in vitro and in vivo models is necessary to assess therapeutic potential and safety.

## Figures and Tables

**Table 1:** Concentration of lentiviral preparations across different batches.

	**Concentration (pg/mL)**
FAP-CAR LV1	1027634.46
FAP-CAR LV2	1833834.37
FAP-CAR LV3	1129229.21

**Table 2:** LNP synthesis prescription.

	**DOTAP**	**DSPC**	**Cholesterol**	**PEG**
Molar ratio	6	15	10	0.62
Quality (mg)	5.0	14.0	4.5	2.0

## Data Availability

The data supporting the findings of this study are available from the corresponding author upon reasonable request.

## Supporting Information

Supporting Information Additional supporting information can be found online in the Supporting Information section. Figure S1: Standard curve for the HIV-1 p24 antigen ELISA kit, demonstrating a high correlation with an *R*^2^ value of 0.99541, indicating excellent assay linearity (*R*^2^ > 0.95). Figure S2: (A) Western blot analysis showing the total expression of CAR proteins in HEK-293T cells following lentiviral transduction (*n* = 3 for each group; ⁣^∗∗∗^*p* < 0.001). (B) Schematic representation of CAR nucleic acid sequence translation into protein fragments using SnapGene. (C) Flow cytometry analysis of CAR expression on the surface of transduced HEK-293T cells (blank, blank control group; control, CAR staining control group; isotype, isotype control group; transduction, transduction group). Figure S3: The lentivirus overexpressing FAP contains a puromycin resistance gene, enabling the selection of FAP-293T stable cell lines. HEK-293T cells were cultured with varying puromycin concentrations to determine the optimal dose. Complete cell death occurred at concentrations of 3 *μ*g/mL or higher, establishing 3 *μ*g/mL as the optimal concentration for stable cell line selection. Figure S4: (A) Western blot analysis of fibrosis marker proteins in MCFs under different TGF-*β* concentrations (*n* ≥ 3 for each group). (B) Immunofluorescence detection of FAP and *α*-SMA in MCFs after 48 h of 10 ng/mL TGF-*β* treatment (*n* ≥ 3 for each group; ⁣^∗^*p* < 0.05, ⁣^∗∗^*p* < 0.01, and ⁣^∗∗∗^*p* < 0.001). Figure S5: (A) ELISA assays measured cytokine secretion (IFN-*γ* and IL-6) after coculture (*n* = 3 for each group). (B) Target cell lysis quantified by LDH release assay at indicated E:T ratios (*n* = 3 for each group). (C) Merged bright-field and green fluorescence images reveal clustering of CAR-Jurkat cells around FAP-293T cells (green). (D) Confocal microscopy showing CAR-Jurkat cells (red, CAR; blue, DAPI) specifically clustering around FAP-293T cells (green, FAP; blue, DAPI) (⁣^∗^*p* < 0.05, ⁣^∗∗^*p* < 0.01, and ⁣^∗∗∗^*p* < 0.001, ns represents no statistical difference).
