# Supplementary material for: Fibroblast Activation Protein-Targeted CAR-T Cells Induce Apoptosis in Murine Cardiac Myofibroblasts
Source: Cardiovasc Ther. 2025 Sep 13;2025:7230505. doi: 10.1155/cdr/7230505 (PMC12450108; doi:10.1155/cdr/7230505)
Supplement: Supporting Information — Additional supporting information can be found online in the Supporting Information section. Figure S1: Standard curve for the HIV-1 p24 antigen ELISA kit, demonstrating a high correlation with an R2 value of 0.99541, indicating excellent assay linearity (R2 > 0.95). Figure S2: (A) Western blot analysis showing the total expression of CAR proteins in HEK-293T cells following lentiviral transduction (n = 3 for each group; ⁣∗∗∗p < 0.001). (B) Schematic representation of CAR nucleic acid sequence translation into protein fragments using SnapGene. (C) Flow cytometry analysis of CAR expression on the surface of transduced HEK-293T cells (blank, blank control group; control, CAR staining control group; isotype, isotype control group; transduction, transduction group). Figure S3: The lentivirus overexpressing FAP contains a puromycin resistance gene, enabling the selection of FAP-293T stable cell lines. HEK-293T cells were cultured with varying puromycin concentrations to determine the optimal dose. Complete cell death occurred at concentrations of 3 μg/mL or higher, establishing 3 μg/mL as the optimal concentration for stable cell line selection. Figure S4: (A) Western blot analysis of fibrosis marker proteins in MCFs under different TGF-β concentrations (n ≥ 3 for each group). (B) Immunofluorescence detection of FAP and α-SMA in MCFs after 48 h of 10 ng/mL TGF-β treatment (n ≥ 3 for each group; ⁣∗p < 0.05, ⁣∗∗p < 0.01, and ⁣∗∗∗p < 0.001). Figure S5: (A) ELISA assays measured cytokine secretion (IFN-γ and IL-6) after coculture (n = 3 for each group). (B) Target cell lysis quantified by LDH release assay at indicated E:T ratios (n = 3 for each group). (C) Merged bright-field and green fluorescence images reveal clustering of CAR-Jurkat cells around FAP-293T cells (green). (D) Confocal microscopy showing CAR-Jurkat cells (red, CAR; blue, DAPI) specifically clustering around FAP-293T cells (green, FAP; blue, DAPI) (⁣∗p < 0.05, ⁣∗∗p < 0.01, and ⁣∗∗∗p < 0.001, ns represents no [file 7230505.f1.docx]

**Supplementary information**


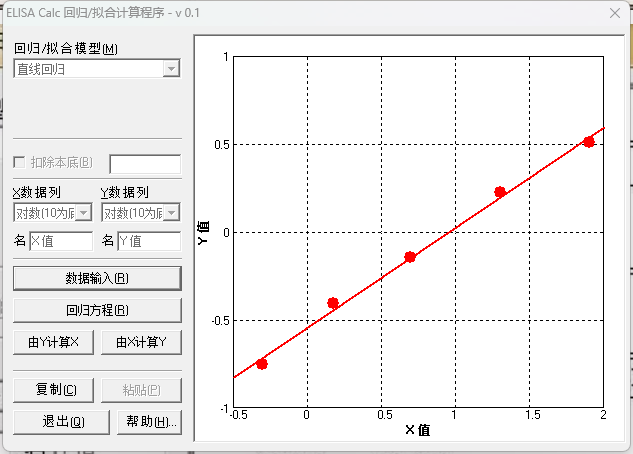


y=-0.54458+0.56942x


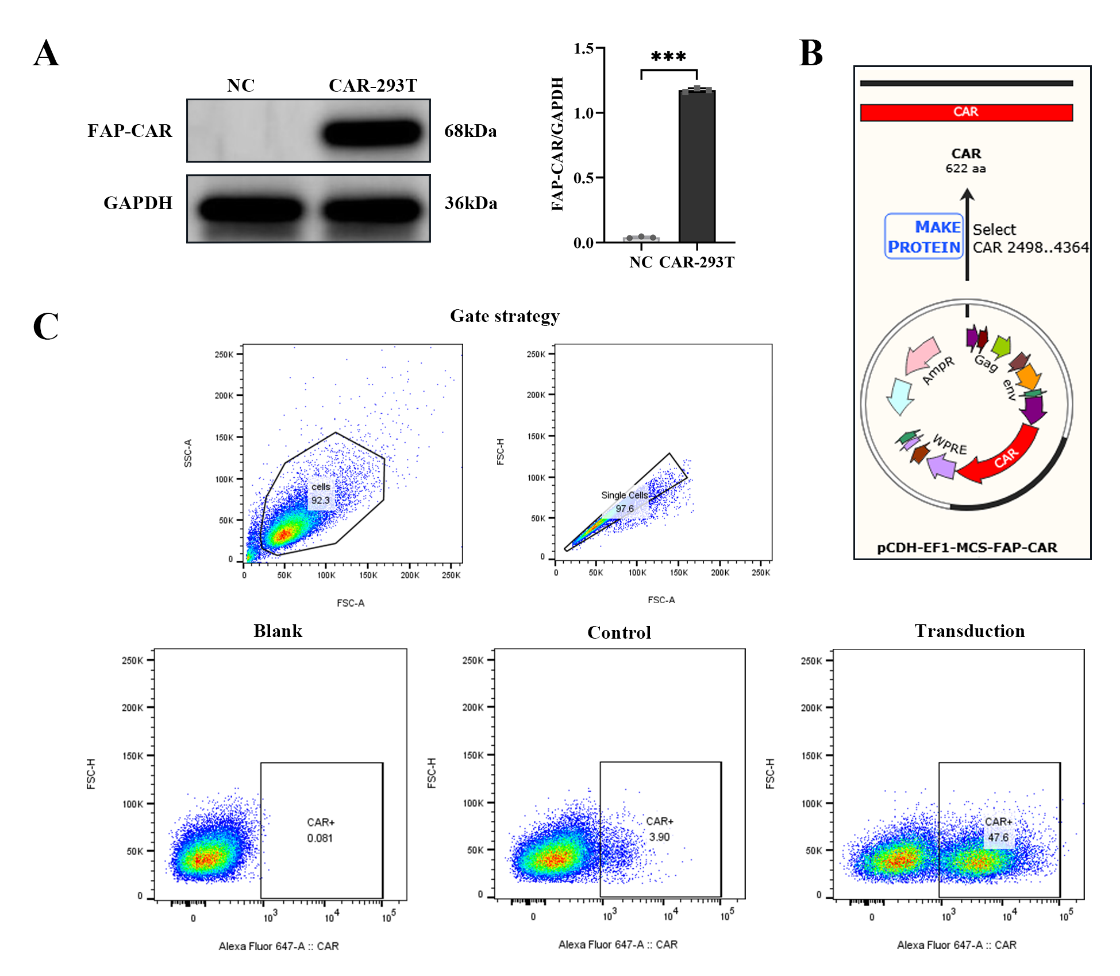
Supplementary Fig. 1. Standard curve for the HIV-1 p24 antigen ELISA kit, demonstrating a high correlation with an R² value of 0.99541, indicating excellent assay linearity (R² > 0.95).

Supplementary Fig. 2. (A) Western blot analysis showing the total expression of CAR proteins in HEK-293T cells following lentiviral transduction (n = 3 for each group , ****p* < 0.001); (B) Schematic representation of CAR nucleic acid sequence translation into protein fragments using SnapGene; (C) Flow cytometry analysis of CAR expression on the surface of transduced HEK-293T cells (Blank: blank control group, Control: CAR staining control group, Isotype: isotype control group, Transduction: transduction group).


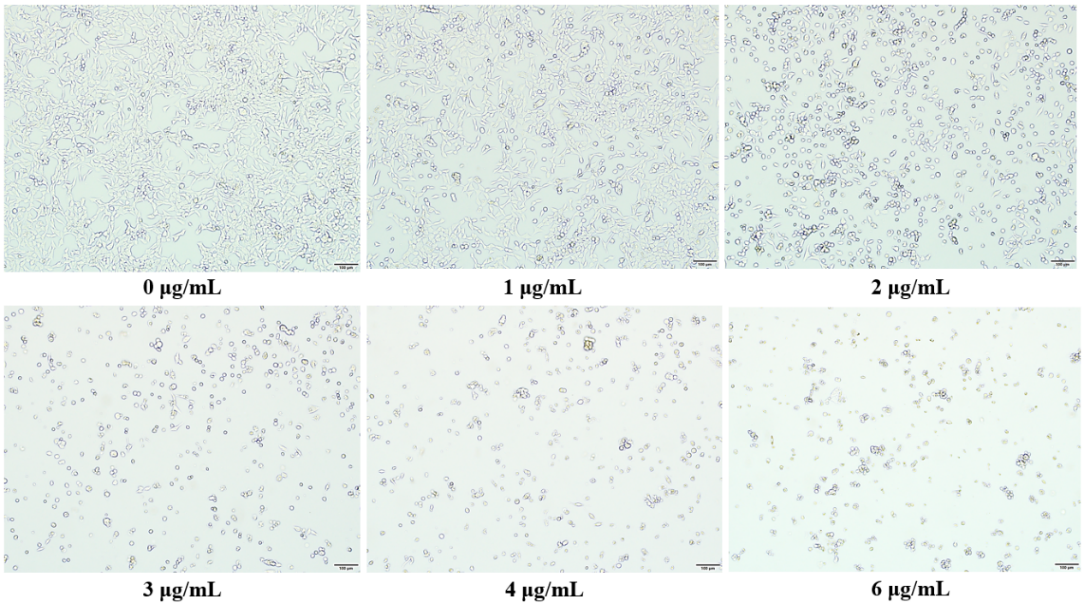


Supplementary Fig. 3. The lentivirus overexpressing FAP contains a puromycin resistance gene, enabling the selection of FAP-293T stable cell lines. HEK-293T cells were cultured with varying puromycin concentrations to determine the optimal dose. Complete cell death occurred at concentrations of 3 μg/mL or higher, establishing 3 μg/mL as the optimal concentration for stable cell line selection.


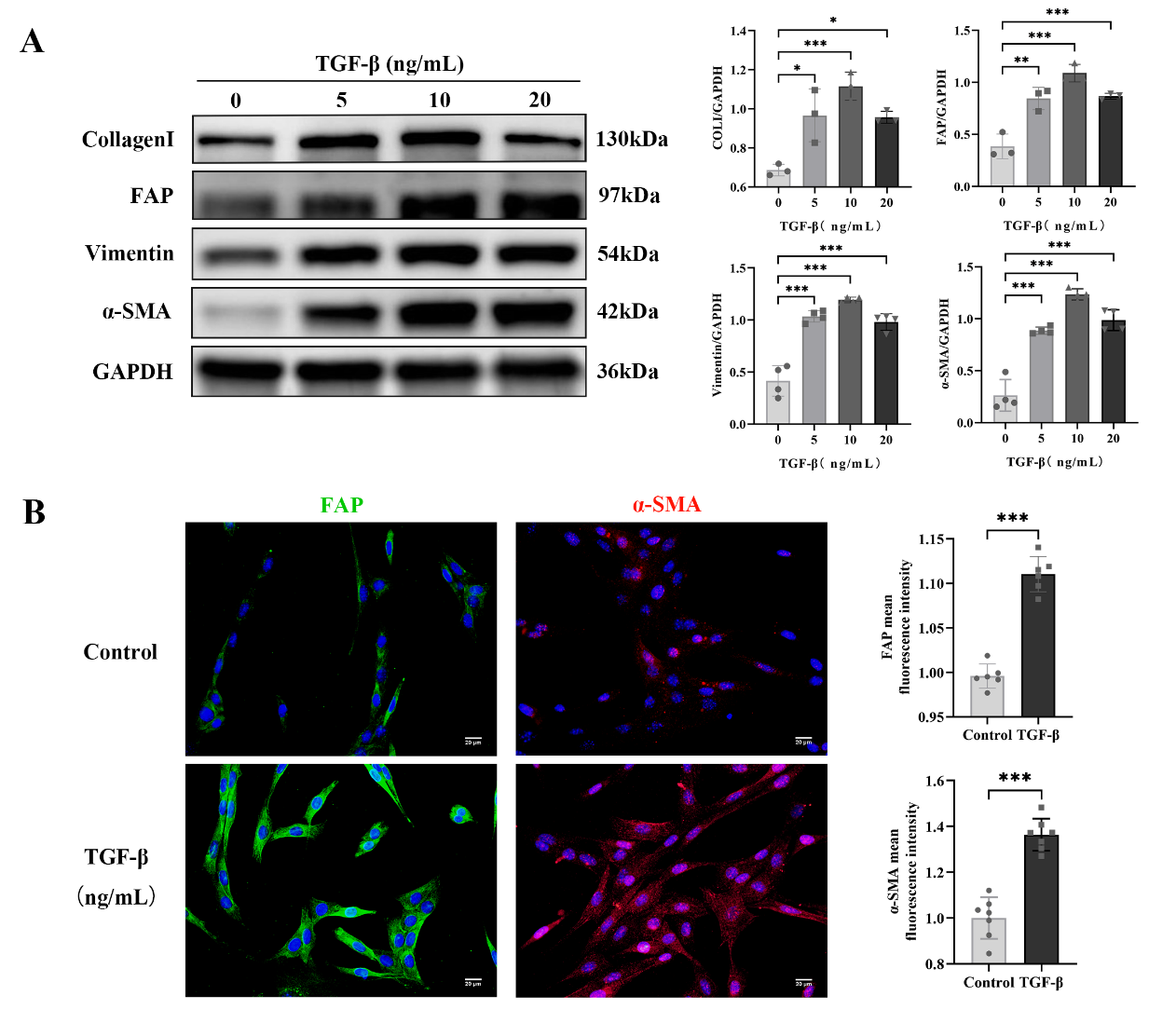
Supplementary Fig. 4. (A)Western blot analysis of fibrosis marker proteins in MCFs under different TGF-β concentrations(n ≥3 for each group); (B) Immunofluorescence detection of FAP and α-SMA in MCFs after 48 hours of 10ng/mL TGF-β treatment (n ≥ 3 for each group. (**p*<0.05, ***p*<0.01, ****p*<0.001)


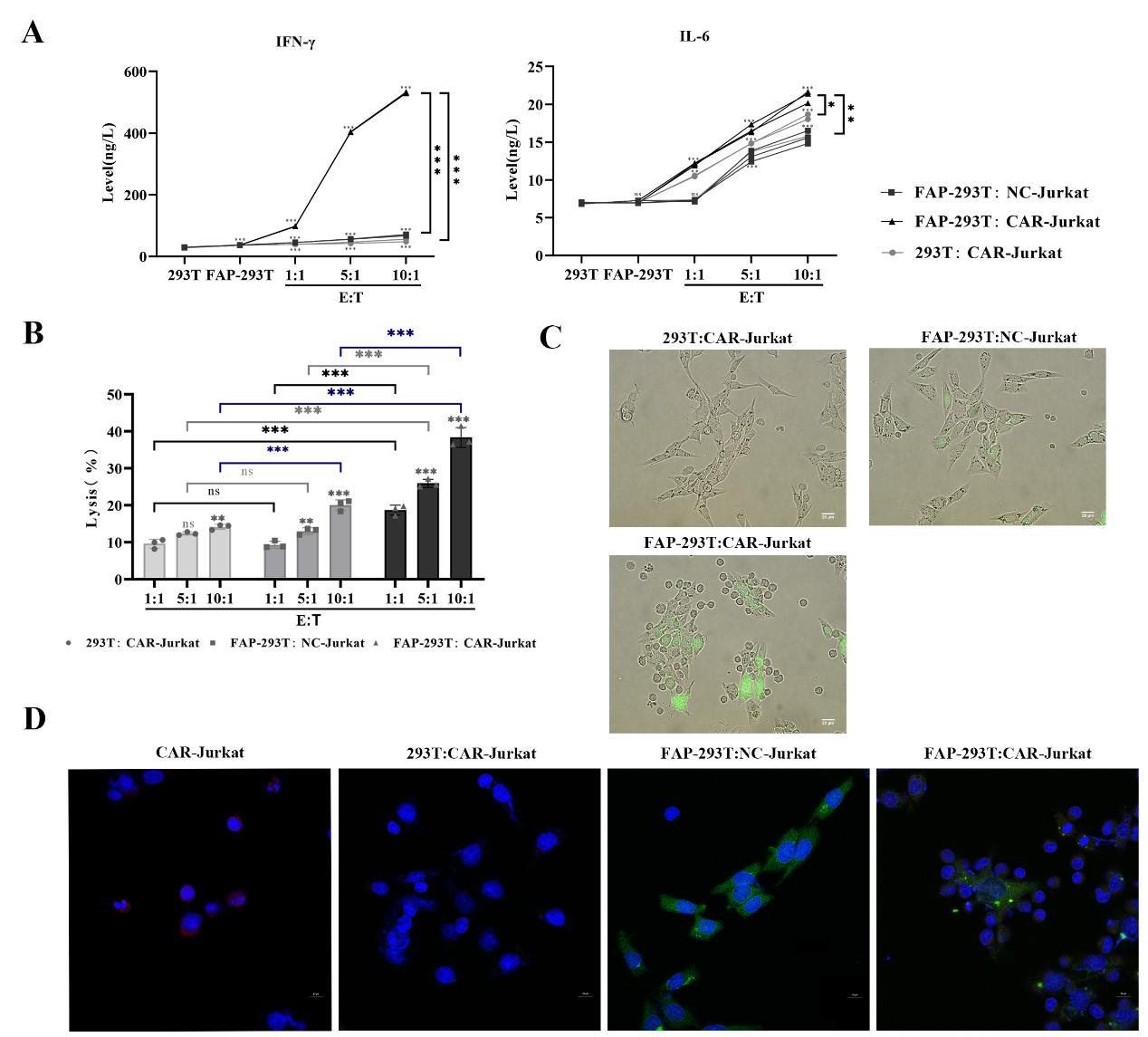


Supplementary Fig. 5. (A)ELISA assays measured cytokine secretion (IFN-γ and IL-6) after co-culture (n = 3 for each group); (B) Target cell lysis quantified by LDH release assay at indicated E:T ratios (n = 3 for each group); (C) Merged bright-field and green fluorescence images reveal clustering of CAR-Jurkat cells around FAP-293T cells (green); (D) Confocal microscopy showing CAR-Jurkat cells (red, CAR; blue, DAPI) specifically clustering around FAP -293T cells (green, FAP; blue, DAPI). (**p*<0.05, ***p*<0.01, ****p*<0.001, ns represents no statistical difference)
